# Phase-Conversion Stiffened Dual-Network Hydrogel for Fracture Plugging in Oil-Based Drilling Fluid

**DOI:** 10.3390/gels11080635

**Published:** 2025-08-12

**Authors:** Xinying Cui, Chengwen Wang, Weian Huang, Shifeng Zhang, Haiqun Chen, Bo Wu

**Affiliations:** 1 School of Petroleum Engineering, China University of Petroleum (East China), Qingdao 266580, China; 2 School of Petroleum and Natural Gas Engineering, Changzhou University, Changzhou 213164, China; 3 Sinopec Huadong Petroleum Engineering Co. Ltd., Nanjing 210000, China

**Keywords:** phase-conversion, dual network, stiffened hydrogel, fracture plugging, oil-based drilling fluid

## Abstract

During drilling operations, lost circulation frequently occurs, leading to significant loss of drilling fluids which causes environmental damage and increasing drilling costs. To address the problem of fracture plugging, gel materials have emerged as an ideal solution due to stable physicochemical properties and excellent environmental compatibility. However, most existing gels exhibit poor stability and low mechanical strength under high-temperature conditions. To overcome these limitations, high-temperature-resistant phase-conversion stiffened dual-network hydrogel for oil-based drilling fluids was developed. Phase-conversion was realized by immersing synthesized double-network hydrogel in ethylene glycol (EG), polyethylene glycol (PEG), and glycerol (Gly), optimizing and enhancing its mechanical properties, followed by plugging performance evaluations. Experimental results demonstrated that the phase-conversion stiffened gels achieved significantly improved compressive strength and plugging efficiency at elevated temperature. The GC-MS results indicated that dehydration and reagent exchange occurred during immersion, with change in the solid content of the sample. After being treated by white oil at high temperature, the oil phase almost replaced the water phase in the gel. The results of ATR-IR confirmed the formation of hydrogen bonds in the gel. TGA data revealed that PEG enhanced the thermal stability of the gel, EG negatively affected thermal stability, and Gly had negligible influence. The enhancement in gel strength primarily stems from the increase in solid content caused by phase transformation. Dehydration and multiple hydrogen bonds formed between organic reagent molecules and polymer chains in the gel have a synergistic enhancement effect.

## 1. Introduction

Due to notable advantages in lubricity, shale inhibition, wellbore cleaning efficiency, and reservoir protection, oil-based drilling fluids have been widely used during drilling shale oil and gas formations, as well as in high-temperature deep formations and complex offshore drilling operations [1,2,3,4,5,6]. However, lost circulation is a frequently occurring down hole accident during drilling operations, causing loss of drilling fluid and extra economical cost [7,8]. Lost circulation, particularly in large-fracture loss zones, requires developments of more effective lost circulation materials (LCMs). Gels, with their flexibility and shape memory function, exhibit strong self-adaptive sealing capabilities for cracks, making them widely used as lost circulation materials or to reduce water production [9,10,11]. Bai et al. fabricated single-network gel particles reinforced with flexible fibers, demonstrating strong oil-absorption capacity and achieving a fracture sealing pressure resistance of 7.6 MPa at room temperature [12], thereby enabling effective fluid loss control of oil-based drilling fluids (OBDFs) during drilling operations. Yang et al. developed a polymeric organogel via soap-free emulsion polymerization, which reduced fluid loss by 68% [13]. The high strength property of the hydrogel plays a key role in achieving effective plugging performance. In addition, considering that temperatures in some deep reservoirs may exceed 120 °C, a tough gel sample should have good high-temperature resistance. However, due to the high water content, gels undergo hydrothermal degradation under high temperature, and the deconstruction of the chemical structures of the hydrogel leads to a sharp decline in mechanical strength [14,15]. Therefore, exploring approaches to enhance gel performance at elevated temperatures has become an important research direction for improving their high-temperature sealing capabilities [16].

The double-network (DN) hydrogels is composed of two polymers, forming a special network structure, which has the advantages of environmental protection and high mechanical properties [17]. Compared to single-network hydrogels, DN hydrogels exhibit better mechanical properties and stability under extreme conditions [18]. Previous studies showed that the poor strength of the gel is caused by several factors. One reason is the uneven distribution of the polymer network in the gel, which creates weak spots that are prone to cracking under stress. Another is the high water content, accompanied by low solid content, which reduces the mechanical strength of the gel at high temperature [19]. In the field of petroleum engineering, DN hydrogels have good feasibility and prospect in plugging [20].

According to the classic “sacrificial bond concept” proposed by Gong et al. [19], the introduction of additional energy dissipation mechanism can enhance the strength of the gel. To enhance the mechanical performance of polymer gels, phase separation and transition offered a fresh dimension and were adopted as new strategies [21,22]. For DN hydrogels, changing the homogeneous solution environment may induce phase separation [23]. Li et al. investigated the mechanism of suppression of crack advance and delayed antifatigue-fracture in self-healing hydrogels [17]. As a result, the phase separation can endow the hydrogels with high strength and toughness. 

In this study, the mechanical strength and plugging performance of DN hydrogels were enhanced via a phase-conversion approach. The DN hydrogel samples were immersed into ethylene glycol (EG), polyethylene glycol (PEG), and glycerol (Gly), respectively. The dehydration and phase separation caused by the reagent significantly enhanced the inter/intra polymer interactions in the gel and improved mechanical properties. The prepared gel can withstand high-temperature conditions and is engineered for lost circulation control in oil-based drilling fluids.

## 2. Results and Discussion

### 2.1. Swelling Property

**Figure 1:**
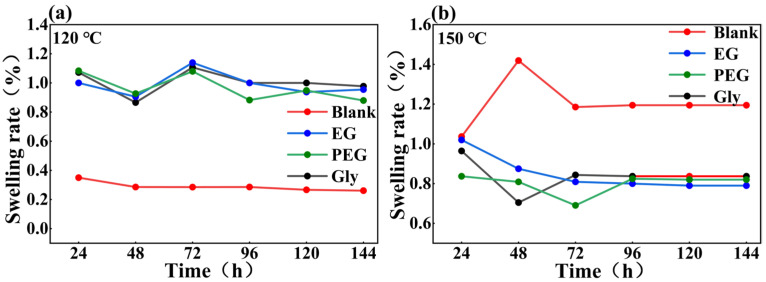
Swelling ratio of hydrogels at different temperatures. (**a**) the samples treated with PEG, EG, and Gly at 120 °C; (**b**) the samples treated with PEG, EG, and Gly at 150 °C.

As shown in Figure 1, the swelling ratio of all samples in white oil increased with the temperature. The swelling ratio of blank sample is about 26% at 120 °C, which proved that the mass of hydrogel decreased rapidly in the hot oil. The shrinkage of gel volume and shape alteration occurred as a result of significant mass loss. The swelling ratio of the samples treated with PEG, EG, and Gly at 120 °C were 88%, 95%, and 97%, respectively (Figure 1a). These results indicated that reagent treatment can improve the swelling property of hydrogel and the mass loss of the gel in white oil at high temperatures can be also effectively reduced. At 150 °C, the swelling ratio of blank sample is about 119%, indicating an increase in the gel’s mass in white oil. This may be due to the high temperature damaging the network structure of hydrogel, allowing extensive infiltration of white oil which resulted in a significant increase in mass. The swelling ratio of the samples treated with PEG, EG, and Gly were 82%, 79%, and 84% at 150 °C, respectively (Figure 1b), indicating that the swelling property of the hydrogel in white oil decreased with increasing temperature. The results showed that liquid-phase-conversion induced by reagent treatment can help improve the morphology and structural stability of the gel at high temperature. The strong capability to maintain the original shape and resist shrinkage significantly contributes to enhanced plugging performance.

**Figure 2:**
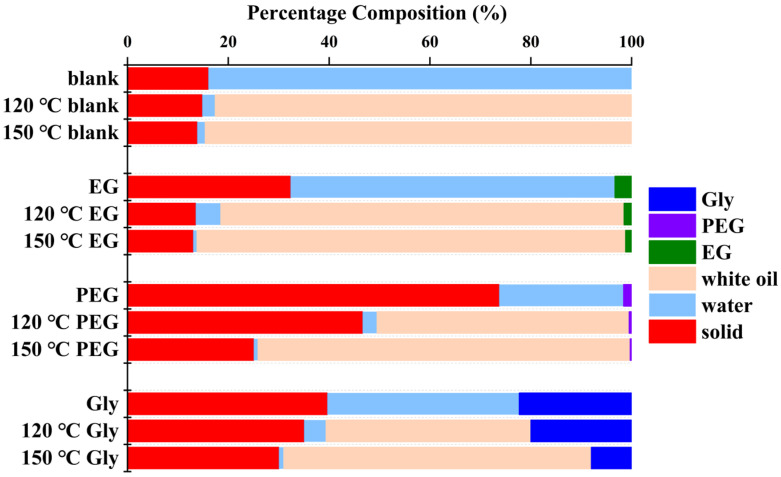
Composition content of gel samples.

Figure 2 showed the percentage composition of gel samples at room temperature and after being treated with high-temperature white oil. According to GC-MS quantification, the solid content of gel samples showed declining trend with increasing temperature. This is due to the fact that the oil phase replaced the water phase at high temperature, and the invasion of white oil increased with the increase in temperature. The invasion of white oil leads to a significant increase in sample volume and mass at elevated temperatures, whereas the mass of solid matter remains constant throughout this process. Consequently, the solid content percentage exhibits a marked decline. Compared to the blank samples, quantitative characterization confirms an increase in solid content for agent-treated DN hydrogels and newly organic components were detected. In reagent-treated samples, Gly demonstrated the highest concentration, indicating superior penetration and diffusion capacity within the gel matrix. The sample treated with PEG showed the highest solid content despite minimal PEG incorporation, suggesting that the observed solid content elevation is primarily attributable to dehydration effects.

### 2.2. ATR-FTIR Spectra Analysis

**Figure 3:**
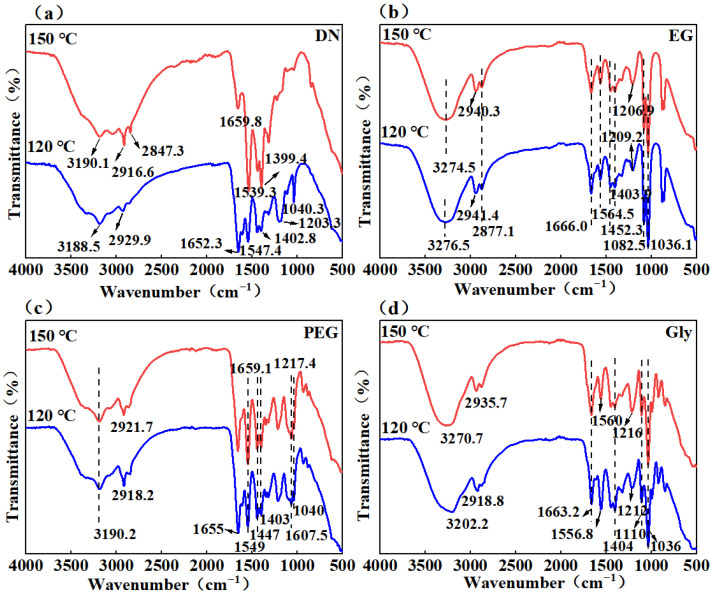
Infrared spectra of hydrogels treated with different reagents: (**a**) Blank sample; (**b**) EG treated sample; (**c**) PEG treated sample; (**d**) Gly treated sample.

The infrared spectra of blank samples and treated samples under two high-temperature conditions were tested. As shown in Figure 3a, in the blank sample, the absorption peaks which attributed to the -NH stretching vibration were observed at 3188 and 3190 cm^−1^ at 120 and 150 °C, respectively. The characteristic absorption peaks of -CH_2_ were found at 2929 and 2916 cm^−1^, and the characteristic absorption peaks of -C=O and -NH were observed at 1652 cm^−1^ and 1659 cm^−1^, respectively, indicating that AMPS and AM monomers formed copolymers through polymerization. The stretching vibration peaks of -NO and -CO at 1203 cm^−1^ and 1040 cm^−1^ at 120 °C were significantly weakened at 150 °C, suggesting that the polymer chains were broken at high temperature. In samples treated with EG (Figure 3b), the absorption peaks at 3274 cm^−1^ and 3276 cm^−1^ correspond to the -OH group, while the peaks at 2940 cm^−1^ and 2941 cm^−1^ are attributed to the -CH_2_ group. Compared to the untreated sample, the C=O stretching peak shifts to 1663 cm^−1^ in glycerol, to 1659 cm^−1^ and 1655 cm^−1^ in PEG, and to 1666 cm^−1^ in EG, indicating the occurrence of hydrogen bonding on the C=O group. After being treated with PEG (Figure 3c), 3190cm^−1^ is the characteristic absorption peak of -OH which was the evidence for the existence of hydrogen bonds. In the sample treated with Gly (Figure 3d), the characteristic absorption peak of -OH was 3270 cm^−1^ at 150 °C, indicating that there are hydrogen bonds between molecules.

### 2.3. Thermal Stability of Hydrogels

**Figure 4:**
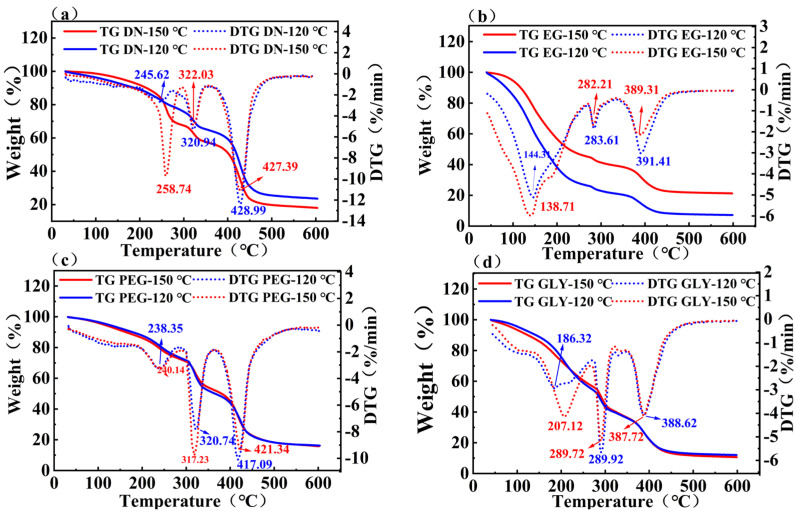
TG and DTG curves of hydrogel samples at 120 ℃ and 150 ℃: (**a**) Blank sample; (**b**) EG treated sample; (**c**) PEG treated sample; (**d**) Gly treated sample.

The thermal stability of hydrogel samples was measured by thermogravimetric analyzer, and the results are shown in Figure 4. The temperature data regarding thermal degradation characteristics were presented in Table 1. TGA curves showed three temperature ranges of hydrogel decomposition, which reflected the composition of the gel. The mass loss of the hydrogel samples below 100 °C can be attributed to the loss of water [24]. There were no T_peak_ below 100 °C in all samples, indicating that the water content was very low, which was consistent with the results of GC-MS (Figure 2). The polymer in the double-network hydrogel will break the molecular chain above 290 °C [25]. As shown in Figure 4a, for blank sample, the first stage is the decomposition stage of water and light components in white oil. The second stage is the decomposition stage of heavy components in white oil. The third stage is the cracking stage of polymer macromolecules. It can be observed that the degradation mass loss of blank sample at 150 °C was higher than 120 °C. The analytical results demonstrated that high-temperature white oil treatment adversely impacts the thermal stability of dual-network (DN) hydrogels. The decreased final residue could result from the increased penetration of white oil at 150 °C. As shown in Figure 4b, for EG sample, the weight loss components of first stage include water, white oil, and EG. Compared with DN gel, the weight loss of EG gel is increased, and the end T_endset_ of stages II and III is decreased, suggesting that EG has a negative effect on the thermal stability of the side and main chains. This is attributed to the higher water content in EG-treated samples (Figure 2), where hydrolysis breaks down the polymer and cross-linking chains, thereby compromising the gel network and reducing thermal stability [26]. The PEG-treated hydrogels clearly showed three pyrolysis stages (Figure 4c). The first stage is the decomposition stage of water, PEG, and light components in white oil. The second stage is the decomposition stage of the polymer side chains and heavy components in white oil. The third stage is the main chain cracking stage of polymer macromolecules. Compared with DN gel, T_endset_ of stages II and III shifted to higher temperature, suggesting that PEG can improve the thermal stability of the side and main chains. As shown in Figure 4d, for Gly samples, stage I is the decomposition stage of water and light components in white oil. Stage II is the decomposition stage of the polymer side chains, heavy components in white oil and Gly. Stage III is the decomposition stage of main chains of polymer. The results showed that, in Gly-treated gel, T_endset_ of stages II and III were similar to that of blank gel, indicating that Gly has a minor impact on the thermal stability of gels.

### 2.4. Compression Strength and Breakthrough Pressure Test

**Figure 5:**
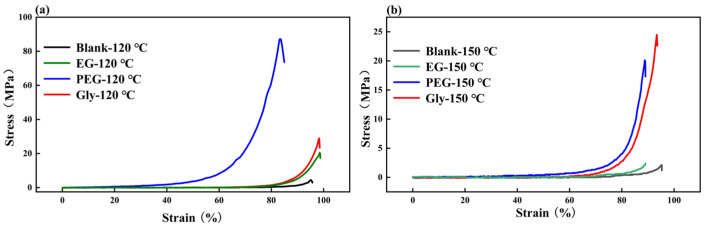
Compressive strength of hydrogel after being treated with different reagents: (**a**) Samples treated with white oil at 120 °C; (**b**) Samples treated with white oil at 150 °C.

**Figure 6:**
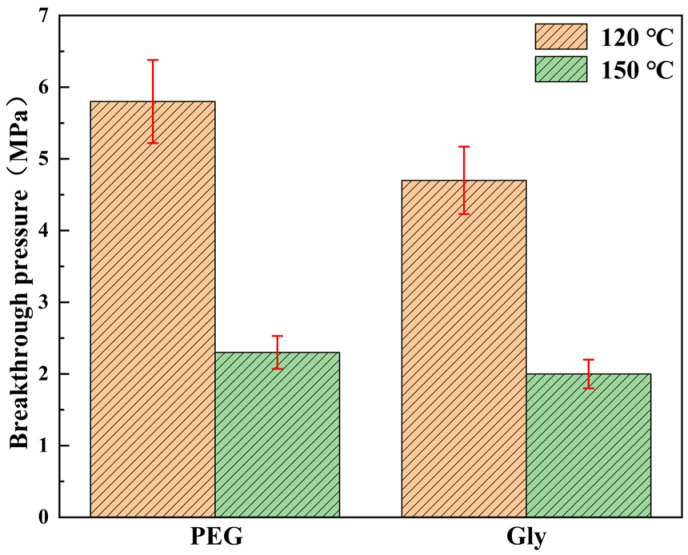
Plugging ability of hydrogel after soaking in different reagents.

As shown in Figure 5, the compressive strength of the phase-conversion stiffened double-network hydrogel samples is significantly improved. In Figure 5a, after immersion in high-temperature white oil at 120 °C, the untreated gel sample exhibited a maximum compressive strength of 4.3 MPa, while the ethylene glycol-treated sample demonstrated a value of 19.9 MPa, representing a 363% improvement. The compressive strength of polyethylene glycol soaking sample is 87.1 MPa, which is increased by 1926%. The pressure bearing value of glycerol soaking sample is 28.9 MPa, which is increased by 572%. In Figure 5b, after soaking in high temperature white oil at 150 °C, the the compressive strength of the unsoaked hydrogel sample was 2.1 MPa, and the value of the ethylene glycol soaked sample was 2.4 MPa, which was increased by 14.29%. The pressure bearing value of polyethylene glycol soaking sample is 20.1 MPa, which is increased by 857%. The pressure-bearing value of glycerol soaking sample is 24.5 MPa, which is increased by 1066%. The test results showed that reagent treatment and the existence of white oil can significantly improve the compressive properties of double-network hydrogels at high temperature. However, the compressive properties of hydrogels decreased with the increase in temperature. The pressure-bearing values of hydrogels without soaking, ethylene glycol soaking, and polyethylene glycol soaking decrease significantly, while the pressure-bearing values of hydrogels soaked in glycerol decrease slightly, indicating that glycerol soaking helps hydrogels to maintain stable compressive properties at high temperature. The compressive strength of the hydrogel will affect the plugging performance. As shown in Figure 6, polyethylene glycol and glycerol soaked hydrogel samples were selected to evaluate the plugging performance of 5 mm cracks at 120 °C and 150 °C due to higher compressive strength. The breakthrough pressure of the sample soaked in polyethylene glycol was 5.8 MPa after being treated with white oil at 120 °C. When the temperature of white oil rises to 150 °C, the breakthrough pressure remains at 2.3 MPa. The breakthrough pressure of the sample soaked in glycerol were 4.7 MPa and 2 MPa after being treated with white oil at 120 °C and 150 °C. The results showed that the phase-inversion hydrogel samples still maintains high-fracture plugging performance at high temperature.

**Figure 7:**
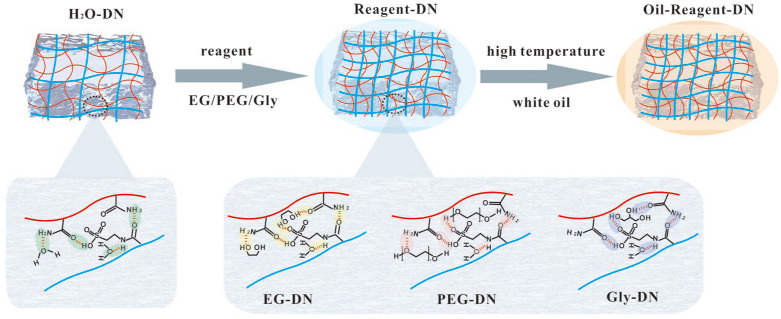
Schematic of the proposed mechanism of reinforcement via phase-conversion (Blue represents the first network and red represents the second network in DN gels).

The possible enhancement mechanism of gel samples was phase-conversion (Figure 7). When the DN gel was immersed in organic reagent, dehydration occurs as free water and bound water within the gel are partly replaced by the reagent, leading to multi-component hydrogel systems. Partial dehydration of the continuous phase resulted in a higher concentration of polymers, which is likely responsible for the increase in solid content. Subsequently, gel samples underwent phase-separation and phase-conversion in white oil due to the coexistence of hydrophilic and hydrophobic substances. Additionally, multiple hydrogen bonds were formed between organic reagent molecules and the polymers in the gel, further enhancing its mechanical properties. As temperature increases, the hydrogen bonding between water molecules and polymer chains weakens, making water molecules more easily displaced by white oil. Additionally, higher temperatures reduce the viscosity of white oil and increase molecular diffusion rates, accelerating its infiltration into the gel. When the phase-conversion gel was immersed in high-temperature white oil, the infiltration of white oil increases with rising temperature, leading to further expulsion of water. This process enhances the solid content of gel samples and results in a denser, more compact structure. Additionally, multiple hydrogen bonds further enhance its mechanical properties.

## 3. Conclusions

In summary, dual-network hydrogel was synthesized with AMPS as the first-network monomer and AM as the second-network monomer via free radical polymerization. The prepared hydrogel samples were then immersed in EG, PEG, and Gly to fabricate phase-conversion stiffened gels. To evaluate the performance of these gels in high-temperature oil-based drilling fluids, swelling tests, compressive strength measurements, and breakthrough pressure tests were conducted on samples treated with high-temperature white oil. The results demonstrated that the phase-conversion stiffened gels exhibit excellent plugging performance in oil-based drilling fluids at high temperature.

Additionally, the samples were characterized using attenuated total reflectance infrared spectroscopy (ATR-IR), thermogravimetric analysis (TGA), and gas chromatography-mass spectrometry (GC-MS). The GC-MS results indicated that dehydration occurred during reagent immersion and high-temperature white oil treatment, while ATR-IR confirmed the presence of hydrogen bonds. TGA data revealed that PEG enhanced the thermal stability of the gel, EG negatively affected thermal stability, and Gly had negligible influence. Therefore, the improvement in mechanical properties of the gel primarily stems from increased solid content due to dehydration and strengthening effects from multiple hydrogen bonds formed between organic reagent molecules and polymer chains within the gel.

Based on the high-temperature resistance in oil-based environments, phase-conversion stiffened dual-network hydrogel can be used for fracture plugging in oil-based drilling fluids. However, the high-temperature enhancement mechanism of phase transition needs more in-depth investigation in the future. Additionally, comprehensive long-term stability assessments of phase transition-enhanced gels in oil-phase environments represent a valuable direction for further research.

## 4. Materials and Methods

### 4.1. Materials 

2-Acrylamino-2-methylpropanesulfonic acid (AMPS), acrylamide (AM), N,N′-Methylenebis (acrylamide) (MBAA), and 2-oxoglutaric acid were provided by Aladdin Biochemical Technology Co., Ltd. (Shanghai Aladdin Biochemical Technology Co., Ltd., Shanghai, China). EG, PEG, and Gly were purchased from Chemical Reagent Co., Ltd. (Yonghua Chemical Co., Ltd., Shanghai, China). Molecular weight of PEG was 200 g/mol. The white oil was purchased from Shenzhen Jieyou Lubricating Oil Co., Ltd. (Shenzhen, China). All chemicals and reagents were used as received.

### 4.2. Development of Dual-Network Hydrogel

By free radical polymerization, first network of hydrogel was prepared with AMPS and MBAA, and the initiator was 2-oxoglutaric acid. Thereafter, the AMPS hydrogel was soaked in solutions containing AM and MBAA, and the second network in the gel was synthesized by the polymerization reaction of AM. The synthesis process of the gel sample was as previously described in detail [26].

### 4.3. Phase-Conversion Induction and Characterization of Gel Samples

The synthesized gel samples were immersed in EG, PEG, and Gly separately. The weight of samples was tested every 6 h after removing the reagents on the surface, and equilibrium was defined when the weight of samples no longer changed. To minimize error, three samples of each type were prepared for repeatability test. The samples were cut into 1 × 1 cm cubes after surface drying and weighting, then placed in an aging tank containing white oil and aged at 120 °C and 150 °C, respectively. After aging for 24 h, 48 h, 72 h, 96 h, 120 h, and 144 h, swelling ratio of samples were calculated by the mass changes of the samples before and after aging [27,28]. Composition content of samples were analyzed by GC-MS using a Thermo 8000 Evo GC–MS instrument (Hangzhou Spectrum Technology Development Co., Ltd., Hangzhou, China). The thermal stability of gel samples was studied by TG test using a platinum Elmer TG4000 thermogravimetric analyzer (Perkin Elmer Enterprise Management (Shanghai) Co., Ltd., Shanghai, China). ATR-IR test of the samples was measured at room temperature using a Nicolet IS50 (Thermo Fisher, Waltham, MA, USA) (Beijing Denoy Fluid Technology Co., Ltd., Beijing, China) infrared spectrometer with a scanning range of 4000 ~ 500 cm^−1^ and a resolution of 4 cm^−1^. The compressive strength test using gel samples of cylinders was carried on a servo-controlled hydraulic loading device (SHM-60T, China) (Chengdu Huakong Mechanical and Electrical Engineering Co., Ltd., Chengdu, China) and the rate of loading was 1 mm/min. The radius of gel samples was 25 mm. The breakthrough pressure test is used to test the plugging performance of the hydrogel at room temperature and the width of the crack mouth is 5 mm, and the width of the end is 0.5 mm [29].

## Figures and Tables

**Table 1:** Temperature data obtained from TGA curves for hydrogel samples.

Sample	Stage	T_onset_ (℃)	T_peak_ (℃)	T_endset_ (℃)	W (%)	Residue (%)
DN-120 °C	I	40	245.62	273.33	21	23.54
	II	273.33	320.94	350	14.06	
	III	350	428.99	468	41.4	
DN-150 °C	I	40	258.74	294.6	33.9	18.06
	II	294.6	322.03	359.1	11	
	III	359.12	427.39	503.3	37.04	
EG-120 °C	I	40	144.31	266.8	55	21.29
	II	266.8	283.61	329	6.7	
	III	329	391.41	487	17.01	
EG-150 °C	I	40	138.71	271	74	7.20
	II	271	282.21	332	5.8	
	III	332	389.31	471	13	
PEG-120 °C	I	40	238.35	286	27	16.27
	II	286	320.74	363	23	
	III	363	417.09	529	33.73	
PEG-150 °C	I	40	240.14	289	28	16.26
	II	289	317.23	366	21	
	III	366	421.34	559	34.74	
GLY-120 °C	I	40	186.32	269	45	12.05
	II	269	289.92	351	20.05	
	III	351	388.62	480	22.9	
GLY-150 °C	I	40	207.12	270	43	12.04
	II	270	289.72	354	22.06	
	III	354	387.72	468	22.9	

## Data Availability

The data presented in this study are openly available in article.
